# Will fencing floodplain and riverine wetlands from feral pig damage conserve fish community values?

**DOI:** 10.1002/ece3.8054

**Published:** 2021-09-24

**Authors:** Nathan J. Waltham, Jason Schaffer

**Affiliations:** ^1^ Centre for Tropical Water and Aquatic Ecosystem Research (TropWATER) College of Science and Engineering James Cook University Townsville Qld Australia

**Keywords:** connectivity, exclusion fences, feral pigs, floodplains, restoration, tropical wetlands

## Abstract

Installation of feral pig (*Sus scrofa*) exclusion fences to conserve and rehabilitate coastal floodplain habitat for fish production and water quality services remains untested. Twenty‐one floodplain and riverine wetlands in the Archer River catchment (north Queensland) were surveyed during postwet (June–August) and late‐dry season (November–December) in 2016, 2017, and 2018, using a fyke net soaked overnight (~14–15 hr) to test: (a) whether the fish assemblage are similar in wetlands with and without fences; and (b) whether specific environmental conditions influence fish composition between fenced and unfenced wetlands. A total of 6,353 fish representing twenty‐six species from 15 families were captured. There were no wetland differences in fish assemblages across seasons, years and for fenced and unfenced (PERMANOVA, Pseudo‐*F* < 0.589, *p* < .84). Interestingly, the late‐dry season fish were far smaller compared to postwet season fish: a strategy presumably in place to maximize rapid disposal following rain and floodplain connectivity. In each wetland, a calibrated Hydrolab was deployed (between 2 and4 days, with 20 min logging) in the epilimnion (0.2 m) and revealed distinct diel water quality cycling of temperature, dissolved oxygen and pH (conductivity represented freshwater wetlands), which was more obvious in the late‐dry season survey because of extreme summer conditions. Water quality varied among wetlands in terms of the daily amplitude and extent of daily photosynthesis recovery, which highlights the need to consider local conditions and that applying general assumptions around water quality conditions for these types of wetlands is problematic for managers. Though many fish access wetlands during wet season connection, the seasonal effect of reduced water level conditions seems more overimprovised when compared to whether fences are installed, as all wetlands supported few, juvenile, or no fish species because they had dried completely regardless of the presence of fences.

## Graphical abstract

Fencing wetlands prevents pig access on tropical floodplains, which should be positive for fish. However, though many fish access (fenced and unfenced) wetlands during wet season connection, the seasonal effect of reduced water level conditions seems to be more overimprovised compared to whether fences are installed or not, as all wetlands supported few, or no fish species because they had dried completed regardless of whether fences were present or not.

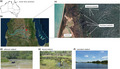

## 1 INTRODUCTION

Wetlands (palustrine and lacustrine) that are located on floodplains away from riverine channels support rich aquatic plant and fauna communities (Ambrose & Meffert, 1999; Brandolin & Blendinger, 2016; Jiang et al., 2015). However, after peak flood connection, aquatic organisms occupying these wetlands face a moving land‐water margin until connection is broken, at which point wetlands have been shown to support a nonrandom assortment of aquatic species, including fish (Arrington & Winemiller, 2006; Pander et al., 2018). The duration, timing and frequency that off channel wetlands maintain lateral pulse connection with primary rivers is an important determining factor in broader contribution to coastal fisheries production—higher floodplain connection results in more fish production is the overwhelming conclusion (Bennett & Kozak, 2016; Galib et al., 2018; Górski et al., 2016; Hurd et al., 2016). In addition to connection, environmental conditions become important on floodplains, including water quality (Waltham & Schaffer, 2018), but also access to shelter to escape predation and available food resources (Blanchette et al., 2014; Jardine et al., 2012). Although optimism about coastal floodplain restoration is building (Waltham et al., 2020), efforts by managers to restore wetland services and values is increasing, though data delineating success are limited. This lack of data becomes important when attempting to quantify biodiversity returns for the funding investment made by government or private investor organizations (Elliott et al., 2016; Waltham & Fixler, 2017; Weinstein & Litvin, 2016; Zedler, 2016).

At some point after floodplain connection, the waters begin receding and progressively disconnect from the main river channel, forming smaller and shallower off channel wetland/swamp refugia (Abbott et al., 2020; McJannet et al., 2014; Pettit et al., 2012; Pusey & Arthington, 2003). In tropical north Australia, formation and persistence of seasonal off channel wetland are more pronounced owing to high evaporation rates, loss to groundwater (Petheram et al., 2008), and in many situations, the water quickly retracts away from the banks and riparian shade (Pusey & Arthington, 2003). After floodplain disconnection from primary rivers, they become more prone to reduced water quality conditions—most notably reduced water depth (Pettit et al., 2012), high water temperatures (Wallace et al., 2017), and suffer extended low oxygen periods (Waltham & Schaffer, 2018). This reduced state of water quality (or habitat) increases aquatic fauna exposure risks to acute and chronic thresholds (Burrows & Butler, 2012; Wallace et al., 2015). In the late‐dry season, fish confined to these isolated wetlands on floodplains therefore have very limited avoidance choices (Waltham & Schaffer, 2018) and must exploit available habitat opportunities (Love et al., 2017; Phelps et al., 2015), which are specific to each wetland depending on orientation and location (Schomaker & Wolter, 2011), depth and vegetation cover (Wallace et al., 2017). Floodplain fish must deal with these vagaries at least until the monsoonal rain again reconnects overbank coastal floodplains.

Across northern Australia, feral pigs (*Sus scrofa*) have been shown to contribute wide‐scale negative impacts on wetland vegetation assemblages, water quality, biological communities, and wider ecological processes (Baber & Coblentz, 1986; Krull et al., 2013). Feral pigs utilize an omnivorous diet supported by foraging or digging plant roots, bulbs, and other below ground vegetation material over terrestrial or wetland areas (Ballari & Barrios‐García, 2014). This feeding strategy has a massive impact on wetland aquatic vegetation communities (Doupe et al., 2010), giving rise to soil erosion and benthic sediment resuspension, reduced water clarity and eutrophication which becomes particularly critical late‐dry season. The fact that limited data exits on the impact that feral pigs contribute to wetlands (Doupe et al., 2010; Marshall et al., 2020; Mitchell & Mayer, 1997; Steward et al., 2018; Waltham & Schaffer, 2018), places a strain on the ability for land managers to quantify the consequences of pig destruction (Commonwealth of Australia, 2017). Conversely, a lack of baseline data means quantifying success following expensive mitigation efforts is problematic (Negus et al., 2019).

Strategies focused on reducing or removing feral pigs from the floodplain landscape have been employed since their introduction to Australia (Fordham et al., 2006). Control strategies have included poison baiting, aerial shooting, and trapping using specially constructed mesh cages (that are baited sometimes) (Ross et al., 2017). Attempts have also included installing exclusion fencing that border the wetland of interest. While advantages of installing fencing around wetlands has been examined only recently in Australia (Doupe et al., 2010), those authors claim fencing might well be less effective particularly in situations where wetlands would normally dry before the next wet season rainfall and reconnection. Fencing is expensive to construct and maintain (Ross et al., 2017), but at the same time prevents other nontarget terrestrial fauna from accessing wetlands, which becomes imperative late‐dry season where wetlands become regional water points for many mobile fauna (Commonwealth of Australia, 2017).

The aims here were twofold: (a) What is the spatial and temporal variability of fish assemblages in waterbodies with and without feral pig fencing, and (b) does this pattern correlate to water quality variables? These data are important and necessary given increasing government funding investment planned in northern Australia for restoration of wetlands impacted by feral animals (including pigs) (Waltham & Schaffer, 2018).

## 2 METHODS

### 2.1 Description of study system

**FIGURE 1:**
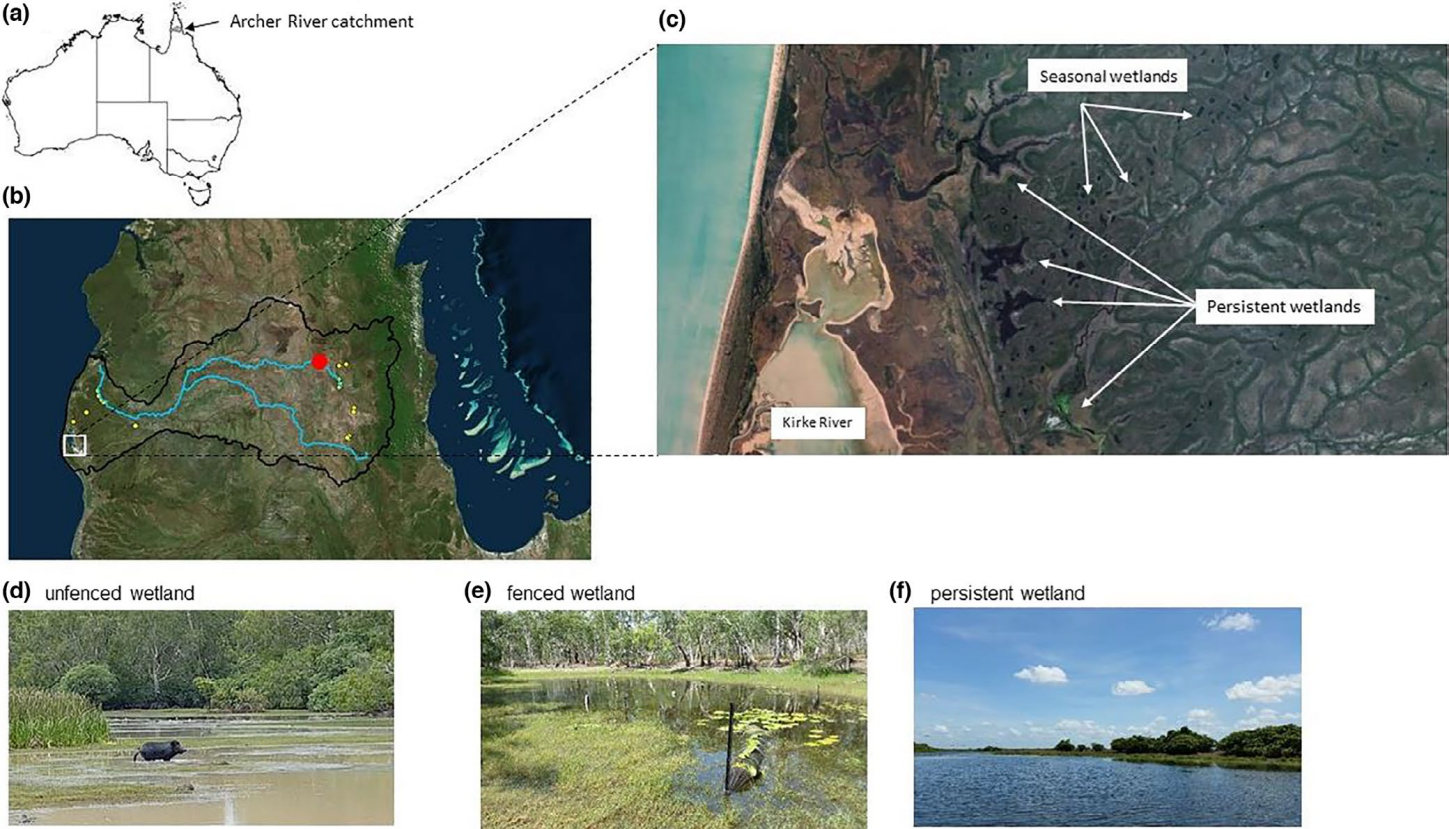
Location of wetlands in this study: (a) location of the Archer River catchment in northern Queensland, Australia and (b) wetland sites on the coastal floodplain and mid‐catchment where feral pig fencing has been completed around wetlands preventing access (yellow circles). The three wetland typologies (c—pig impacted wetlands that are shallow (typically <0.5 m deep), without submerged aquatic vegetation, turbid, and eutrophic; d—fenced wetland preventing pig access that are deeper (typically <2 m deep), clear with submerged aquatic vegetation present) exist across the catchment; and e—permanent wetlands that are deeper (typically <2 m deep), steep sides limiting pig access, clear with submerged aquatic vegetation present. Archer River gauge station (red circle)

The Archer River catchment is located on Cape York Peninsula, north Queensland (Figure 1). The head waters of the river rise in the McIlwraith range on the eastern side Cape York, where it flows and then enters Archer Bay on the western side of the Gulf of Carpentaria; along with the Watson and Ward Rivers. The catchment area is 13,820 km^2^, which includes approximately 4% (510 km^2^) of wetland habitats (https://wetlandinfo.des.qld.gov.au/wetlands/facts‐maps/basin‐archer/), such as estuarine mangroves, salt flats and saltmarshes, wet heath swamps, floodplain grass sedge, herb and tree Melaleuca spp. swamps and riverine habitat. The lower region of the catchment includes part of the Directory of Internationally Important Wetland network (i.e., nationally recognized status for conservation and cultural value) that extends along much of the eastern Gulf of Carpentaria, including the Archer Bay Aggregation, Northeast Karumba Plain Aggregation and Northern Holroyd Plain Aggregation. Two national parks are located in the catchment (KULLA (McIlwraith Range) National Park, and Oyala Thumotang National Park). Land use is predominately grazing with some mining activities planned in the next few years on the northern bank of the river (not within the area of this study).

**FIGURE 2:**
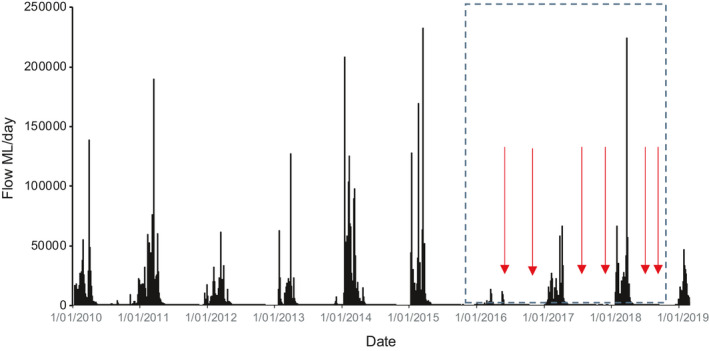
Daily discharge at the Archer River roadhouse gauge (Figure 1) before and during (dashed insert box) this study. Sampling occasions (arrows) are indicated. Data provided by the Queensland Government

Rainfall is tropical monsoonal, strongly seasonal, with between 60% and 90% of total annual rain occurring between November and February. Rainfall records for the catchment reveal highest wet season rainfall occurred in 1989/1999 (2,515 mm), while lowest was 1960/1961 (563.5 mm) (Waltham & Schaffer, 2017). Total antecedent rainfall for the wet season prior (Nov 2014 to Feb 2015) to this survey was 1,081 mm, which is below the 10th percentile for historical records. The wet seasons experienced through the years prior to this study (2010–2015) were among the wettest on record, within the 95th percentile of the long‐term data records. The low rainfall during this study may have contributed to a short flood duration, and thereby connection between wetlands and the main Archer River, when compared to average or above average rainfall years where connection is presumed to be far longer (Figure 2).

Twenty‐one wetlands were sampled including both floodplain and riverine wetlands that were not on the main flow channels, but on anabranches and flood channels that connect to the main channels only during high flow conditions. All wetlands have been historically damaged by pigs (and cattle to a lesser extent) for up to 160 years (Gongora et al., 2004; Lopez et al., 2014), and there is no background data on the wetland condition before introduction of feral pigs in the region. In response to the obvious and widespread impact in the region, a small number were fenced to prevent feral pig and cattle from accessing wetlands, in accordance with the feral animal research and management program (to meet the objectives of traditional owners in the region) of both Kalan enterprises and Aak Puul Ngangtam, and their partners.

The characteristics of each wetland are summarized in Table S1. Here, sampling focused on two periods: (a) immediately following the wet season after disconnection between the river and wetlands (hereafter referred to as postwet season); and (b) late‐dry season (hereafter late‐dry) in 2016, 2017 and 2018. Each sampling campaign was completed over 14 days with six total campaigns (postwet and late‐dry season in 2016, 2017, and 2018).

### 2.2 Field methods

In each wetland, a calibrated high frequency Hydrolab multi‐parameter logger (OTT Hydromet USA) was deployed (0.2 m depth) for between 2 and 4 days to record epilimnion (0.2 m) water temperature, dissolved oxygen (%), electrical conductivity, and pH every 20 min ‐ logging at this frequency provides explicit insight into diel changes in environmental water processes (Wallace et al., 2015, 2017). Weather conditions were fine with all surveys occurring on the falling limb of the hydrograph.

Fish were collected in wetlands using a fyke net (0.8 m opening, double 4 m wing panels, 1 mm stretch mesh) that was soaked overnight (approximately 14:00–09:00). Wetlands substantially impacted by feral pigs; secchi disk depth <0.1 m, no submerged or floating aquatic plants exist, while the fenced wetlands were generally deeper (up to 1.5 m), and had submerged aquatic vegetation (Figure 1). Fish were placed in a tub (~150 L) temporarily, identified, measured (standard length, mm), and returned to the wetland alive in accordance with Australian laws (except for a small number that were kept for food web studies, data not shown here).

### 2.3 Data analysis

There are two main biases in the sampling method here: (a) that the technique will capture large numbers of schooling fish along the wetland margins; and (b) the fact that predatory aquatic fauna including fish, snakes (macleays watersnakes, *Pseudoferania polylepis*), file snakes (*Acrochordus arafurae*)), and freshwater turtles (*Chelodina rugosa*, *Chelodina canni,* and *Emydura s. worrelli*) were periodically trapped for hours means that they could consume fish caught in nets. To overcome these uncertainties, analyses were based on presence/absence of species. Presence/absence provide robust data when relative abundance are of doubtful validity because it deals with species with a diversity of behaviors, trophic functions, and spatial distribute in a more equivalent way than fully quantitative techniques (Quinn & Keough, 2002).

Multivariate differences were examined using PERMANOVA using the Bray–Curtis similarities measure (Clarke, 1993) with significance determined from 10,000 permutations of presence/absence transformation. Multivariate dispersion were tested using PERMDISP, however, homogeneity of variance could not be stabilized with transformation, and therefore, untransformed data were used. Three factors where included years (fixed), season (fixed); and fenced/unfenced (random). These factors were determined *a‐prior* during study design—in addition, the 2016 late‐dry season only had a single fenced wetland site; this data point was removed in the PERMANOVA. Spatial patterns in multivariate fish assemblage structure and the importance of explanatory data sets were analyzed using a multivariate classification and regression tree (mCARTs) (De'Ath, 2002) package in R (version 3.4.4). Analysis was conducted using presence/absence transformed fish data for the 10 species that occurred in >20% of wetland sites (to remove rare species). Selection of the final tree model was conducted using 10‐fold cross‐validation, with a 1‐*SE* tree; the smallest tree with cross‐validation error within 1 *SE* of the tree with the minimum cross‐validation error (Sheaves & Johnston, 2009). The relative importance of the explanatory variables were assessed to determine those with a high overall contribution to tree node split, with the best overall classifier being given a relative importance of 100%.

Kolmogorov–Smirnov (K‐S) two‐sample tests determined differences in the overall shape of fish body size distribution using a Bonferroni correction for multiple comparisons. K‐S tests take into account differences between the location, skew, and kurtosis of frequency distributions, but do not identify which of these parameters are driving distributional differences. Therefore, we report the following characteristics of each body size distribution to further describe any differences found: mean, standard deviation (*SD*), minimum value (min), maximum value (max), the range of values, skewness, and kurtosis.

## 3 RESULTS

### 3.1 Hydrology and wetland water quality

Wet season rainfall totals in the Archer River catchment were low during the study period compared to the preceding years (Figure 2), with rainfall within the 10th percentile for historical recordings held by the Australian Bureau of Meteorology. This means that some caution is necessary with interpretation of these data, namely that floodplain connectivity under higher rainfall years is likely to have a longer duration when compared to lower connection duration under the current rainfall conditions.

A full summary of water quality data are provided in Supplementary files (S1). In summary, water temperatures during the study period were generally about 26℃ (Table 1). Minimum water temperature recordings as low as 18℃, while maximum temperatures occurred in November 2016 survey reached above 40℃ for several hours of the day in some instances. The water column exhibited pronounced diel temperature periodicity; 1 or 2 hr after sunrise each day. Near‐surface water temperatures began to rise at an almost linear rate for a period of 8.0 ± 0.5 hr, generally reaching daily maxima during the middle of the afternoon. The mean daily temperature amplitude was 6.2°C (highest daily amplitude 9.6°C, lowest 4.4℃). For the remaining 16 hr of each day, near‐surface water temperatures gradually declined reaching minimum conditions shortly after sunset.

**TABLE 1:** Fish taxa identified in the Archer River (freshwater section) from broad northern Australia survey of freshwaters, and those species presented in wetlands recorded during this study

Family	Genus	Species	Common name	Present in Archer River	Present in wetlands
Apogonidae	*Glossamia*	*aprion*	Mouth almighty	√	√
Ariidae	*Neoarius*	*berneyi*	Berney's catfish	√	
	*Neoarius*	*graeffei*	Lesser salmon catfish	√	
	*Neoarius*	*leptaspis*	Triangular shield catfish	√	
	*Neoarius*	*paucus*	Silver cobbler	√	
Atherinidae	*Craterocephalus*	*stercusmuscarum*	Fly‐speck hardyhead	√	√
Belonidae	*Strongylura*	*krefftii*	Long tom	√	√
Centropomidae	*Lates*	*calcarifer*	Barramundi^a^	√	
Chandidae	*Ambassis*	sp.	Glass perch	√	√
	*Ambassis*	sp.	Northwest glassfish	√	
	*Ambassis*	*agrammus*	Sailfin glassfish	√	
	*Ambassis*	*elongatus*	Elongate glassfish	√	
	*Ambassis*	*macleayi*	Macleay's glassfish	√	√
	*Denariusa*	*bandata*	Pennyfish	√	√
Clupediae	*Nematalosa*	*erebi*	Bony bream	√	√
Dasyatidae	*Dasyatis*	sp.	Freshwater stingray^b^	√	
Eleotridae	*Hypseleotris*	*compressa*	Empire gudgeon		√
	*Mogurnda*	*mogurnda*	Northern purple‐spot gudgeon	√	√
	*Oxyeleotris*	sp.	Gudgeon	√	
	*Oxyeleotris*	*nullipora*	Poreless cod	√	
	*Oxyeleotris*	*lineolatus*	Sleepy cod	√	√
	*Oxyeleotris*	*selheimi*	Giant cod	√	√
Engraulidae	*Thryssa*	*scratchleyi*	Freshwater anchovy	√	
Gobiidae	*Glossogobius*	*aureus*	Golden goby	√	
	*Glossogobius*	*giuris*	Flathead goby	√	
	*Glossogobius*	sp2	Goby (Munroi)	√	
	*Glossogobius*	sp3	Goby (Dwarf)	√	
Megalopidae	*Megalops*	*cyprinoides*	Oxeye herring	√	√
Melanotaeniidae	*Iriatherina*	*werneri*	Threadfin rainbowfish	√	√
	*Melanotaenia*	*nigrans*	Black‐banded rainbowfish	√	√
	*Melanotaenia*	*splendid inornata*	Chequered rainbow fish	√	√
	*Melanotaenia*	*trifasciata*	Banded rainbow fish	√	√
	*Melanotaenia*	sp.	Rainbowfish	√	
	*Pseudomugil*	*tenellus*			√
	*Pseudomugil*	*gertrudae*			√
Osteoglossidae	*Scleropages*	*jardinii*	Saratoga	√	√
Plotosidae	*Anodontiglanis*	*dahli*	Toothless catfish	√	
	*Neosilurus*	sp.	Eel‐tailed catfish	√	
	*Neosilurus*	*ater*	Black catfish	√	√
	*Neosilurus*	*hyrtlii*	Hyrtl's tandan	√	√
	*Porochilus*	*rendahli*	Rendahl's catfish	√	√
Pristidae	*Pristis*	*pristis*	Freshwater sawfish^b^	√	
Soleidae	*Synaptura*	*salinarum*	Freshwater sole	√	
Synbranchidae	*Ophisternon*	sp.	Swamp eel	√	^c^
Terapontidae	*Amniataba*	*percoides*	Banded grunter	√	√
	*Hephaestus*	*carbo*	Coal grunter	√	
	*Hephaestus*	*fuliginosus*	Sooty grunter	√	
	*Leiopotherapon*	*unicolor*	Spangled perch	√	√
	*Scortum*	*ogilbyi*	Gulf grunter	√	
Toxotidae	*Toxotes*	*chatareus*	Archer fish	√	√
	*Toxotes*	*jaculatrix*	Banded archerfish	√	
Total species				48	26

^a^ Denotes species of economic importance.

^b^ Denotes species declared as endangered under Australian conservation and biodiversity legislation.

^c^ Swamp eel caught in macroinvertebrate samples.

The electrical conductivity (EC) was very low (Table S1) during the postwet season surveys, while the late‐dry season conductivity was higher, a consequence of evapo‐concentration. The lowest wetland in the catchment (AR08 located on the coastal floodplain) recorded the highest conductivity, suggesting connection with tidal water from the nearby estuary at some stage.

**FIGURE 3:**
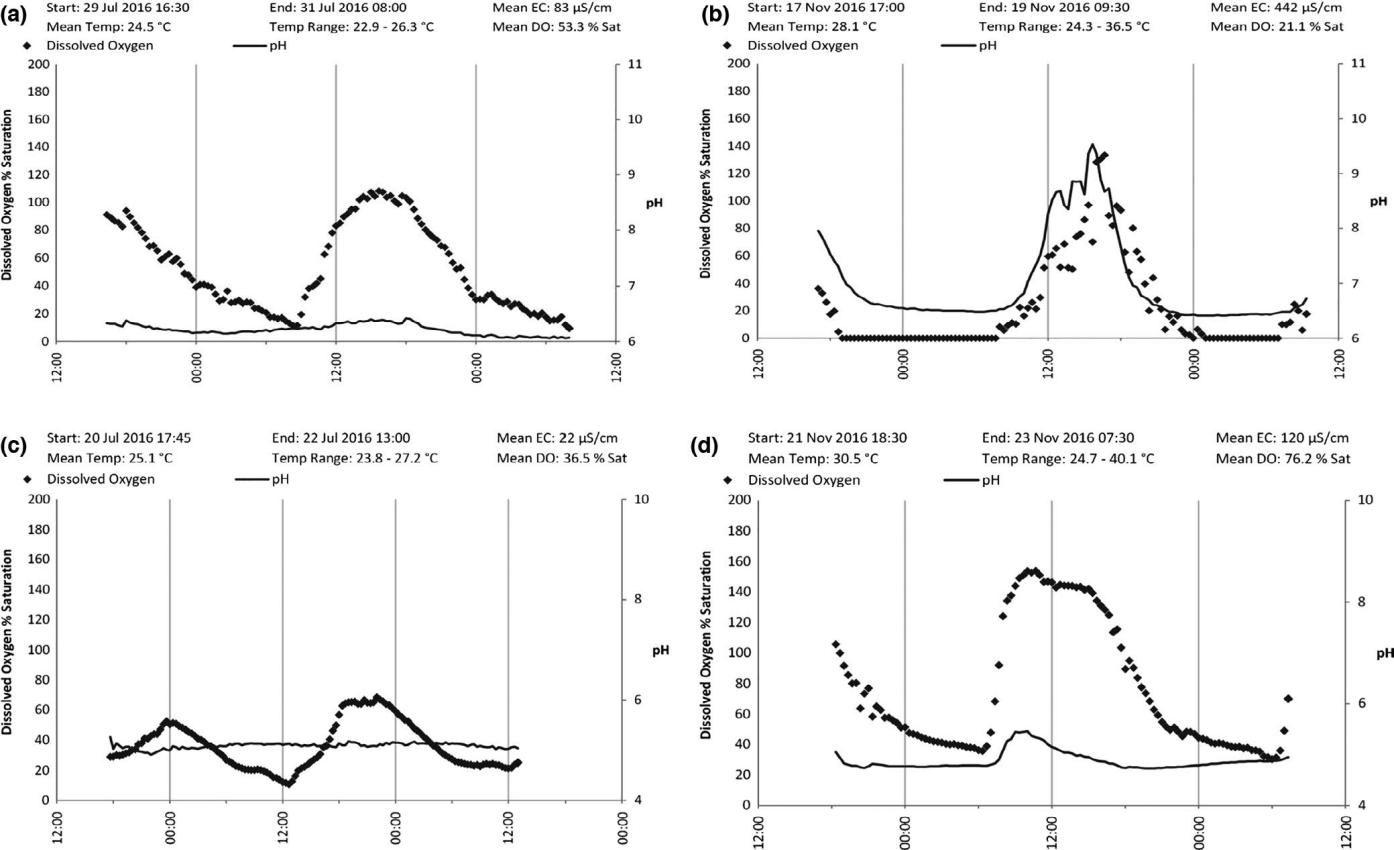
Examples of the diel dissolved oxygen, pH, water temperature, and conductivity cycling in Archer River wetlands. These examples are from KA06 during postwet season (a), and late‐dry season (b) in 2016, and AR01 during postwet season (c) and late‐dry season (d) in 2016

There was evidence of cyclical daily DO fluctuations supporting the contention that biological diel periodicity processes were probably not significantly inhibited in all wetlands (Figure 3). Daily minimum DO concentrations were low enough to suggest there was enough respiratory oxygen consumption to measurably affect water quality, particularly so at the pig impacted wetlands, but also during the late‐dry season survey in November 2016. Dissolved oxygen (DO) seemed to reach daily minima conditions, well below the asphyxiation thresholds of sensitive fish species, in the early morning hours during all surveys. In the examples shown, after the morning low DO (following overnight respiration processes), conditions generally recovered to approximately 50%, but reaching a high of 100%–160% in the late afternoon (before sunset).

pH is also potentially subject to the same kinds of biogenic fluctuations as DO, due to consumption of carbon dioxide (i.e., carbonic acid) by aquatic plants and algae during the day (through photosynthesis), and net production of carbon dioxide at night. If respiratory oxygen consumption is predominant, DO concentrations are low and pH values are generally moderately acidic to neutral, which was the case for wetlands examined here. All photosynthetically active organisms’ utilize carbon dioxide as a preferred carbon source. Some species (including most green algae) are unable to photosynthesize if carbon dioxide is unavailable, but there are other species (including most cyanobacteria and submerged macrophytes) which can utilize bicarbonate as an alternative carbon source. Carbon dioxide consumption causes pH to rise to values in the order of 8.6–8.7 (but that was not the case here during this survey period).

### 3.2 Fish community

A total of 6,353 fish were captured, representing twenty‐six species from 15 families (Table 1). The most common species was the freshwater glassfish (*Ambassis* sp., 51% total catch), delicate blue‐eyes (*Pseudomugil tenellus*, 11%), and northern purple‐spot gudgeon (*Morgunda morgunda*, 9%). A greater number of fish species were caught in the postwet season survey, with a lower number captured during the late‐dry season, including the northern purple‐spot gudgeon (*Morgunda mogunda*), chequered rainbow fish (*Melanotaenia s. inornata*), and the empire gudgeon (*Hypseleoptris compressa*). In addition to fish, we captured a freshwater crayfish (*Cherax* sp.), macleays watersnakes (*Pseudoferania polylepis*) and freshwater turtles (*Chelodina rugosa* and *Emydura s. worrelli*) in most wetlands, notably during postwet season. Overall, there was no significant difference among seasons, fenced/unfenced wetlands, and years (PERMANOVA, Pseudo‐*F* < 0.589, *p* < .84).

**FIGURE 4:**
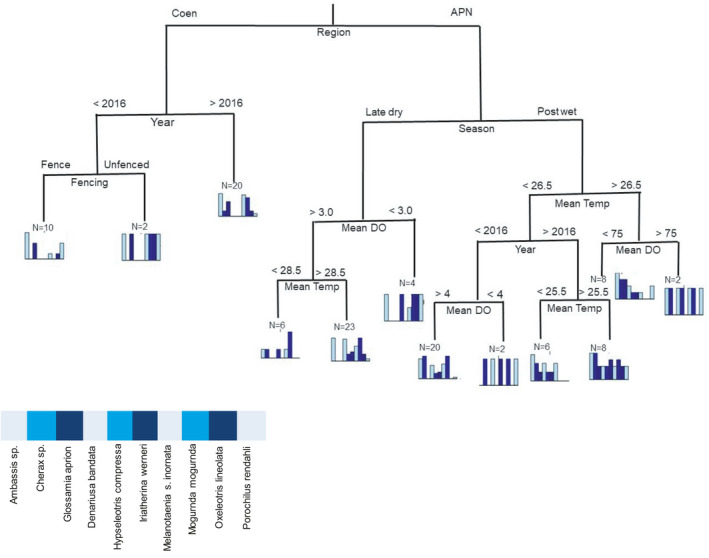
Multivariate regression tree showing the major divisions in the database on assemblage composition. Each of the splits are labeled with the contributing variable, and the division threshold (in the case of electronic conductivity; EC, and dissolved oxygen; DO). The length of the descending branches is proportional to the divergence between groups. Bar plots represent the fish assemblage composition at the corresponding color code node sharing the same attributes. Values in the bar plots represent the relative frequencies of occurrence of each taxon within a same node

With a reduced list confined to dominant species, occurrence profiles for groups in the terminal branches of the mCART analysis (Figure 4) show two initial wetland groups based on a split supported by region, with wetlands in the Coen (mid‐catchment) region separating from those wetlands in the coastal plains. Following the left branch there is interannual variation among wetlands, and a second terminal node based on whether wetlands were fenced in 2016, but not so in 2017 and 2018 data. Following the right branch (APN, coastal plains), the first node separates seasons, and following late‐dry season wetlands further separate based on mean dissolved oxygen (~3.0%), and then mean temperature (~28.5℃). The postwet season branch appears to have more separation among data, with a separation based on mean water temperature (~26.5℃), years, and then finally dissolved oxygen (~4%).

Mean fish body size distributions differed between the three sample years (with fish for each wetland and survey pooled) (KS, *p* < .001, Tables S2‐S5), with larger fish measured in 2017 (50.5 mm) compared to 2016 (38.7 mm) and 2018 (31.6 mm), despite the assemblages having similar size ranges. When comparing the overall fish size distribution by pooling years, postwet season fish were larger (44.9 mm) when compared to the late‐dry season (39.7 mm) (KS, *p* < .01). For some fish species such as the chequered rainbow fish (*Melanotaenia s. inornata*), the postwet season (32.5 mm) was similar when compared to late‐dry season (38.4 mm) (KS, *p* = .06, S3). In contrast, the northern purple‐spot gudgeon (*Mogurnda mogurnda*) was larger postwet season (52.8 mm) compared to late‐dry season (37.1 mm) (KS, *p* < .01, Table S4).

## 4 DISCUSSION

While installation of fences can protect terrestrial ecosystem services from feral impacts (Bariyanga et al., 2016), in the case here fences appear to offer little overimprovised fish additional value compared to those that are not fenced—overall the fish assemblage remained similar across years, seasons, and with and without fencing. While this is the case, importantly what this means is that many fish indeed access both fenced and unfenced wetlands during wet season connection, however, the seasonal effects of reduced water level conditions and the loss of fish assemblage as the dry season progresses is a pattern that remains regardless of fencing. To this end, installation of expensive exclusion fences might not offer additional protection to fish species habitat on this tropical floodplain. The same conclusion was reported by Doupe et al. (2010) where those authors surveyed strongly seasonal wetlands (similar to the wetlands here) elsewhere in northern Australia, and concluded that the seasonal dry down of wetlands ultimately prohibits the wetland contribution to future year successful fish recruitment. In contrast, where floodplain wetlands remain more permanently connected, fish can take more advantage of rich food and nutrient‐rich floodplains (Hurd et al., 2016; Love et al., 2017).

The low species richness in wetlands relative to the main Archer River channel might be a consequence of the frequency and duration of connection between wetlands and the main Archer River. The wet season rainfall immediately prior, and during this survey, was within the 10th percentile for historical records. In research elsewhere, a longer connection duration was shown to result in more fish present postwet season, and conceivably, more species present late‐dry season (Arthington et al., 2015; Hurd et al., 2016). Examples exist where longer connection between main river channels and wetlands contributes positively to fish growth rates, and higher abundance and diversity of fish (Barko et al., 2006; Love et al., 2017; Schomaker & Wolter, 2011). It is also possible that the field methods used here confound our ability to determine the full species composition in wetlands—this could be overcome by using additional survey techniques, including multipanel gill nets, traps, or electrofishing (though we attempted to electrofish these wetlands, and however, the conductivity was too low to effectively use that method), in addition to the presence of crocodiles in these wetlands present a real challenge to sampling. Future research might consider riparian vegetation condition, benthic, and floating aquatic plant extent and pig impact pressure as potential correlating variables describing the fish assemblage in fenced and unfenced wetlands.

An obvious characteristic of the fish assemblage here were larger, presumably adult, individuals in the wetlands after the wet season compared to small individuals present in the late‐dry season. This suggests that the wetlands serve as important refugia for successful recruitment of freshwater fish that adult fish remaining in the wetlands after disconnection are able to complete imperative life cycle stages. The fact that we did not catch large fish in the late‐dry season suggests that adult fish might be lost as the dry season progresses, consumed either by predators such as estuarine crocodiles (*Crocodylus porosus*). Wetlands are also popular feeding and roosting locations for water birds (Brandolin & Blendinger, 2016; Chacin et al., 2015); we observed a large number of species at most wetlands in the late‐dry season. The value of wetlands to wader birds is limited by the condition (Robertson et al., 2017; Żmihorski et al., 2016), but are thought to provide an important nutrient subsidy more broadly on seasonal floodplains (Buelow et al., 2018; Ma et al., 2010). Hurd et al. (2016) postulates that differences in fish communities between main channel and off channel waters is more influenced by the presence of piscivorous predators, or even via a function of competitive exclusion within fish guilds as resources diminish as the late‐dry season takes hold. Examining this point could be achieved by investigating the species niche width (Jackson et al., 2011; Swanson et al., 2015) in drying waters by constructing food webs in individual waters to determine species ranges and changes with fencing treatment, and comparing postwet season and late‐dry season conditions.

In the late‐dry season for the few fish species present, juveniles dominated the catch regardless whether wetlands were fenced. Having small recruits in the late‐dry period might be an important strategy in maximizing dispersal after connectivity with the commencement of the wet season (Pusey et al., 2018). Moreover, late season conditions with no flow and warm conditions might favor larval development (Godfrey et al., 2016; King et al., 2003). *Melanotaeniid* rainbowfish, for example, have a flexible reproductive behavior that is well adapt to deal with the vagaries of temporal variation in habitat conditions (Pusey et al., 2001). The same is true for both *Eleotrid* gudgeon species here with smaller recruits presumably ready for wide‐scape distribution with the pending wet season flow. Pusey et al. (2018) provide a case that the reproduction success of freshwater fish in northern Australia could in fact hinge on antecedent flow patterns across the landscape, and that this flexibility ensures population level success (Stewart‐Koster et al., 2011). This strategy might be particularly appropriate given the below average summer rainfall totals seen during this survey, particularly when compared to previous years.

**FIGURE 5:**
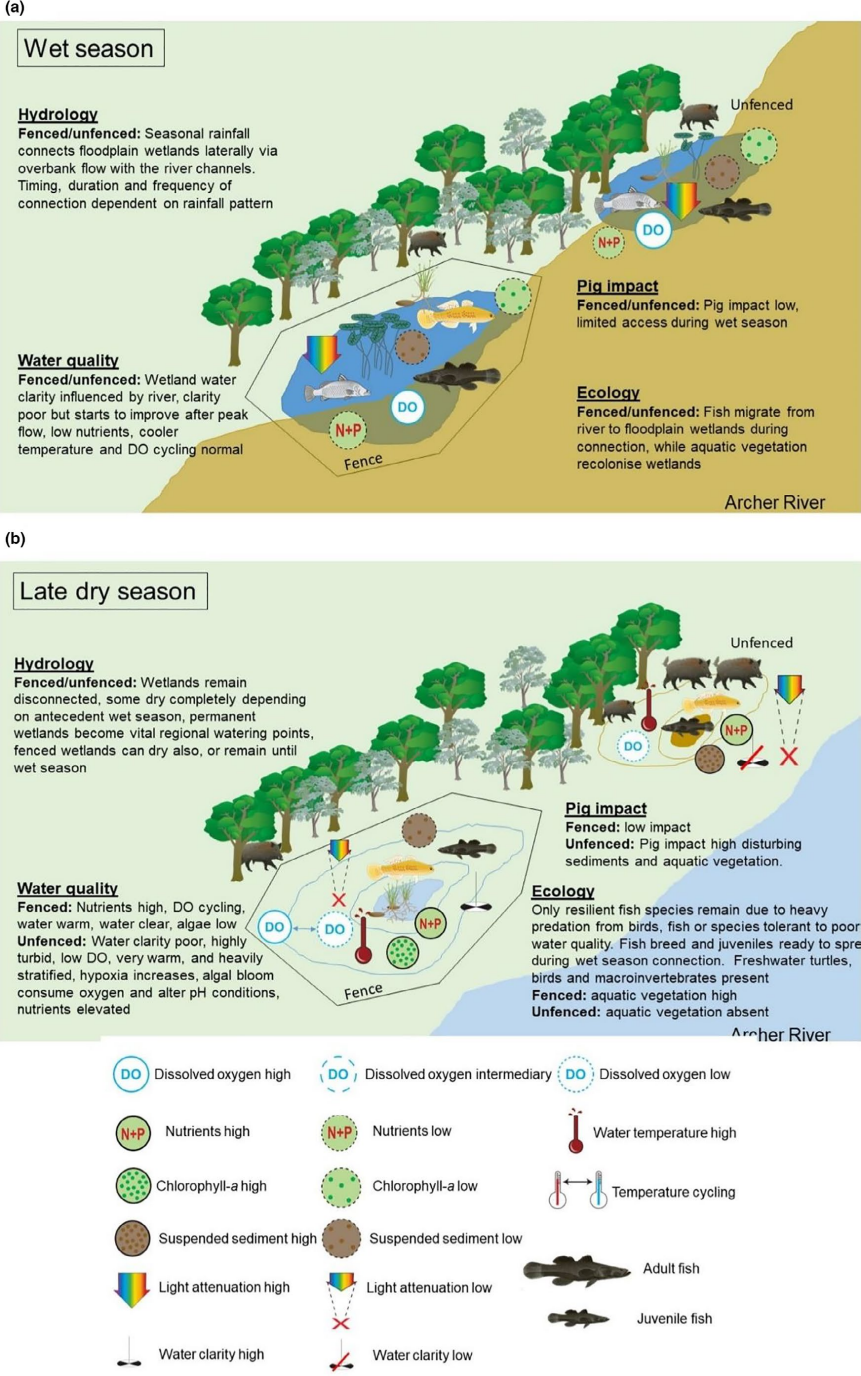
Conceptual diagram of wetland ecosystem conditions during (a) wet season, and (b) late‐dry season. During the wet season, the lateral connection between the Archer River channel and wetlands occurs, during which fish can access wetlands and water quality is generally best because feral pig impact is minimal regardless of fencing. The dry season results in water retracting from the land margins, allowing pigs to access unfenced wetlands. At this stage, water quality conditions are poor in unfenced wetlands with high turbidity/nutrients and temperature, and dissolved oxygen is generally critical for fish. Fenced wetlands become shallower too, through temperature and dissolved oxygen cycling reduced, turbidity is low (<20 NTU), while nutrients can be also high (>20 NTU). Regardless of fencing, fish community reduced to a few resilient species dominated by juveniles ready for rapid dispersal when wet seasons commences again

As the dry season takes hold, water quality conditions progressively deteriorate mostly because of increasing impact from rooting pigs as they access the wetland vegetation. Generally, fenced wetlands change little in terms of water conditions (Figure 5). However, it is the late‐dry season when water conditions are poorest and therefore most critical to fish. Unfenced wetlands tended to be shallower, highly turbid, and suffer water temperatures that exceed acute thermal effects thresholds for fish—which does provide good justification for fencing wetlands, particularly those that are more permanent, such as those spring feed, compared to wetlands that will dry because they are so distant from the primary water course (Waltham & Schaffer, 2018). The most critical water quality condition for fish survival is dissolved oxygen, and the solubility of dissolved oxygen in water is strongly affected by temperature (i.e., high temperature reduces dissolved oxygen solubility (Diaz & Breitburg, 2009). Data on hypoxia tolerances of local freshwater fish species in northern Queensland are available (Butler & Burrows, 2007), and while tolerances vary between species and life stages, there were obvious periods in wetlands when these threshold limits are exceeded. During critical periods, fish must regulate breathing either via increasing ventilation rates (Collins et al., 2013), or by rising to the surface to utilize aquatic surface respiration and/or air gulping (e.g., tarpon, *Megalops cyprinoides*). In any case, the capacity for fish to do that safely depends on the timing of the oxygen sag and antecedent conditions, though notably it appears that most hypoxia‐induced fish kills originate from thermal stress and sunburn resulting from the animals' need to remain at the surface during the heat of the day in order to access available oxygen for respiration. Increasing these risks to fish can have important chronic effects including reducing physical fitness to successfully contribute to future populations (Flint et al., 2018; Gilmore et al., 2018).

## 5 SUMMARY AND CONCLUSIONS

The cultural and ecological value of coastal wetlands means that management intervention is increasingly necessary to ensure they remain productive and viable habitat (Creighton et al., 2015; Canning & Waltham, 2021). Overall, these data support a model that damage to wetlands from pig activities not only contributes to reduced aquatic habitat, through loss of aquatic vegetation communities, but also probably has secondary impacts including water temperature and asphyxiation risks for many hours each day, that are higher than when compared to fenced wetlands (Figure 5). However, fish occupying fenced and unfenced wetlands here were similar, particularly in the late‐dry season where those remaining few species were small and presumably juveniles ready for wet season redistribution. On this basis, installing fences to both floodplain and riverine wetlands that were not on the main flow channels, but rather were on anabranches and flood channels that connect to the main channels only during high flow conditions, seems to offer little additional habitat value for fish from the treat of feral pig impact. Where wetlands are largely ephemeral and will dry anyway, or where wetlands remain until the next seasons rain connection; species abundance and/or diversity is not improved by restricting feral pig access—the exception is that unfenced wetlands tend to be hotter and experience lower available oxygen for fish which may support fencing wetlands most distant from primary water courses if they are like to remain until the next wet season. Further research is necessary to examine climate change resilience on permanent wetlands (and managed wetlands) particularly whether they provide a similar level of refugia as future climate warming in the region is likely to result in more variable wet season rainfall and flow patterns (James et al., 2017). Under this scenario, it is possible that even the more persistent wetlands might suffer similar dry out fate to the ephemeral wetlands examine here.

## CONFLICT OF INTEREST

The authors declare that the research was conducted in the absence of any commercial or financial relationships that could be construed as a potential conflict of interest.

## AUTHOR CONTRIBUTIONS

**Nathan J. Waltham:** Conceptualization (lead); data curation (lead); formal analysis (lead); funding acquisition (lead); investigation (lead); methodology (lead); project administration (lead); resources (lead); validation (lead); visualization (lead); writing‐original draft (lead); writing‐review and editing (lead). **Jason Schaffer:** Investigation (supporting); methodology (supporting); validation (supporting); writing‐review and editing (supporting).

## Supporting information

Tables S1‐S4

## Data Availability

All data associated with this publication (sample sites, coordinates, environmental data and fish catch) can be accessed via https://doi.org/10.5061/dryad.hqbzkh1gv.
