# Supplementary material for: Will fencing floodplain and riverine wetlands from feral pig damage conserve fish community values?
Source: Ecol Evol. 2021 Sep 24;11(20):13780–92. doi: 10.1002/ece3.8054 (PMC8525148; doi:10.1002/ece3.8054)
Supplement: Supplementary file 1 — Tables S1‐S4 [file ECE3-11-13780-s001.docx]

**Supplementary notes**

**Table S1.** Near surface water quality results for high frequency logging at sites. Mean water temperature, electrical conductivity, pH and dissolved oxygen (%); full data series available in the Supplementary. Management intervention included, along with year and survey season. PW – post wet season; LD – late-dry season. Turbidity (NTU) – Low < 20ntu; High > 20ntu.

**Table S2.** Body size distribution for fish species caught in 2016 and 2017 pooled across surveys (post wet and late-dry season)

|  | 2016 | 2017 | 2018 |
| --- | --- | --- | --- |
| N | 302 | 1090 | 629 |
| Mean (mm) | 38.74 | 50.45 | 31.64 |
| Median (mm) | 32.0 | 32.0 | 25.0 |
| SD | 25.427 | 58.28 | 28.97 |
| Min (mm) | 1 | 10 | 11 |
| Max (mm) | 160 | 570 | 371 |
| Range (mm) | 159 | 560 | 360 |
| Skewness | 2.67 | 4.173 | 7.658 |
| Kurtosis | 8.413 | 22.454 | 75.518 |

**Table S3.** Body size distribution for fish species caught in post wet season and late-dry season pooled across years

|  | Post wet season | Late-dry season |
| --- | --- | --- |
| N | 1213 | 808 |
| Mean (mm) | 44.92 | 39.74 |
| Median (mm) | 30.0 | 30.0 |
| SD | 54.79 | 33.67 |
| Min (mm) | 1 | 10 |
| Max (mm) | 570 | 371 |
| Range (mm) | 569 | 361 |
| Skewness | 4.649 | 4.936 |
| Kurtosis | 27.419 | 36.533 |

**Table S4.** Body size distribution for *Mogurnda mogurnda* caught in post wet season and late-dry season pooled across years

|  | Post wet season | Late-dry season |
| --- | --- | --- |
| N | 153 | 260 |
| Mean (mm) | 52.78 | 37.06 |
| Median (mm) | 52.0 | 32.0 |
| SD | 21.89 | 16.32 |
| Min (mm) | 17 | 15 |
| Max (mm) | 110 | 95 |
| Range (mm) | 93 | 80 |
| Skewness | 0.502 | 1.497 |
| Kurtosis | -0.590 | 1.684 |

**Table S5.** Body size distribution for *Melanotaenia s. inornata* caught in post wet season and late-dry season pooled across years

|  | Post wet season | Late-dry season |
| --- | --- | --- |
| N | 72 | 156 |
| Mean (mm) | 32.51 | 38.35 |
| Median (mm) | 30.50 | 35.50 |
| SD | 11.920 | 12.904 |
| Min (mm) | 15 | 18 |
| Max (mm) | 70 | 110 |
| Range (mm) | 55 | 92 |
| Skewness | 1.086 | 2.593 |
| Kurtosis | 1.541 | 11.740 |
